# Peripheral non-enzymatic antioxidants as biomarkers for mood disorders: Evidence from a machine learning prediction model

**DOI:** 10.3389/fpsyt.2022.1019618

**Published:** 2022-11-07

**Authors:** Yuandong Gong, Zhe Lu, Zhewei Kang, Xiaoyang Feng, Yuyanan Zhang, Yaoyao Sun, Weimin Chen, Guanglei Xun, Weihua Yue

**Affiliations:** ^1^Shandong Mental Health Center, Shandong University, Jinan, China; ^2^Peking University Sixth Hospital, Peking University Institute of Mental Health, Beijing, China; ^3^National Clinical Research Center for Mental Disorders, Peking University Sixth Hospital, Beijing, China; ^4^NHC Key Laboratory of Mental Health, Peking University, Beijing, China; ^5^PKU-IDG/McGovern Institute for Brain Research, Peking University, Beijing, China; ^6^Chinese Institute for Brain Research, Beijing, China

**Keywords:** non-enzymatic antioxidants, bipolar disorder, major depressive disorder, machine learning model, uric acid

## Abstract

**Background:**

Oxidative stress is related to the pathogenesis of mood disorders, and the level of oxidative stress may differ between bipolar disorder (BD) and major depressive disorder (MDD). This study aimed to detect the differences in non-enzymatic antioxidant levels between BD and MDD and assess the predictive values of non-enzymatic antioxidants in mood disorders by applying a machine learning model.

**Methods:**

Peripheral uric acid (UA), albumin (ALB), and total bilirubin (TBIL) were measured in 1,188 participants (discover cohort: 157 with BD and 544 with MDD; validation cohort: 119 with BD and 95 with MDD; 273 healthy controls). An extreme gradient boosting (XGBoost) model and a logistic regression model were used to assess the predictive effect.

**Results:**

All three indices differed between patients with mood disorders and healthy controls; in addition, the levels of UA in patients with BD were higher than those of patients with MDD. After treatment, UA levels increased in the MDD group, while they decreased in the BD group. Finally, we entered age, sex, UA, ALB, and TBIL into the XGBoost model. The area under the curve (AUC) of the XGBoost model for distinguishing between BD and MDD reached 0.849 (accuracy = 0.808, 95% CI = 0.719–0.878) and for distinguishing between BD with depression episode (BD-D) and MDD was 0.899 (accuracy = 0.891, 95% CI = 0.856–0.919). The models were validated in the validation cohort. The most important feature distinguishing between BD and MDD was UA.

**Conclusion:**

Peripheral non-enzymatic antioxidants, especially the UA, might be a potential biomarker capable of distinguishing between BD and MDD.

## Introduction

Bipolar disorders (BD) and major depressive disorder (MDD) are serious mental disorders that are characterized by diverse clinical symptoms, with a high prevalence, and impose a heavy disease burden (1). Due to the misdiagnosis between BD and MDD, patients with BD often receive an inappropriate treatment, especially for BD starting with a depressive episode, which leads to repeated attacks (2). It is challenging for clinicians to distinguish between BD and MDD based only on their clinical symptoms. Therefore, there is an urgent need to identify a reliable, objective biomarker to differentiate BD from MDD.

Oxidative stress is critical for the normal physiological functions of the human body, but excessive levels of peroxides contribute to deleterious oxidation, consequently leading to various pathological mechanisms. The brain is a lipid-rich organ with enormous oxygen consumption and an insufficient antioxidant barrier, which makes the brain highly susceptible to deleterious oxidation. Such oxidative imbalances are linked to various psychiatric disorders, including MDD and BD (3, 4).

The oxidative stress system is ensured by a complicated antioxidant defense system (5, 6). The main enzymatic antioxidants include superoxide dismutase, catalase, glutathione transferase, and glutathione peroxidase, which play important roles in cells. Non-enzymatic antioxidants constitute the antioxidant defense in extracellular fluid; these compounds include glutathione, certain vitamins, uric acid (UA), albumin (ALB), total bilirubin (TBIL), and some metal ions (7, 8).

Numerous abnormalities in antioxidant defense have been noted in association with mood disorders. Compared with healthy controls, patients with BD were found to have increased glutathione-transferase, catalase, UA, and decreased glutathione (9, 10). MDD was associated with increased superoxide dismutase and decreased UA (11, 12). However, the results of studies on the activity of antioxidants differ significantly, and there are few studies that compared the differences in antioxidant profiles between BD and MDD. Data on UA, ALB, and TBIL are easy to obtain because they are included in the routine bloodwork examinations performed in hospitals. In addition, the three indices, jointly accounting for approximately 85% of the antioxidant capacity of plasma (13, 14), effectively represent the level of peripheral antioxidants.

Extreme gradient boosting (XGBoost) is a gradient-boosting-based algorithm that employs increasing accurate approximations to find the best models and applies the advanced regularization technique. It enhances the model training speed and generalization and reduces the model complexity. However, the XGBoost method has some limitations. For example, XGBoost does not perform well on high-dimensional sparse features or unstructured data; in such a case, there are too many parameters, and the parameter optimization is too complex. In the present study, the XGBoost method is suitable because the clinical data are two-dimensional structured data. We first investigated the differences in non-enzymatic antioxidants between BD and MDD. Then, we assessed the predictive value of non-enzymatic antioxidants in mood disorders by applying a machine learning model, and we also used the logistic regression method to confirm the strength of the XGBoost prediction model.

In the present study, we have two hypotheses. First, we hypothesize that the differences in three peripheral non-enzymatic antioxidants between BD and MDD are significant. Second, we hypothesize that peripheral non-enzymatic antioxidants can efficiently distinguish between BD and MDD.

## Materials and methods

### Participants

All procedures involving human subjects were approved by the Clinical Research Ethics Committee of Shandong Mental Health Center ([2021] (Research) Ethical Review No. [66]), and the protocol was compliant with the Code of Ethics of the World Medical Association (Declaration of Helsinki). Informed written consent was obtained from all participants.

The inclusion criteria for the discovery cohort were as follows: 1) a diagnosis of BD or MDD based on the International Classification of Diseases, 10th version (ICD-10); 2) Having at least one completed test of UA, ALB, and TBIL during the first 3 days of hospitalization. The HCs had no mental disorders and a family history of mental disorders.

Subjects in the validation cohort were chosen from our previous study which has been published (15); the detailed information is shown in the Supplementary materials.

### Data extraction

The data of the discovery cohort were obtained from the electronic medical record system of Shandong Mental Health Center. It included sex, age, diagnosis, and laboratory examination results (including ALB, TBIL, and UA). The discharge diagnosis was defined as the main diagnosis, and all the diagnoses were confirmed by third-level ward rounds (during the first week of hospitalization) according to the criteria of the hospitalization procedure. Any change in diagnosis was recorded, and the discharge diagnosis was consistent with the changed diagnosis. We extracted the first four consecutive test results of ALB, TBIL, and UA of patients with BD and MDD during a hospital stay. All the data were anonymous. During the data extraction, all the data were stripped of personal identifiers and instead labeled with ID numbers; personnel with access to the ID numbers could not identify any of the participants in this study.

### Statistical analysis

The analyses were conducted by using IBM SPSS Statistics, Version 26 (Chicago Inc., USA) and R software. All measurement data were inspected for normality with the Kolmogorov–Smirnov test. A Chi-square test was conducted to analyze the sex distributions. Differences in ALB, TBIL, and UA among the BD, MDD, and HC groups were tested by analysis of covariance (ANCOVA), with age and sex as covariates; a Bonferroni test was used to identify the pairwise differences between groups. A generalized estimation equation was used to compare the different post-treatment trends of non-enzymatic antioxidants between BD and MDD. We used XGBoost to build a machine learning model. Then, we used 10-fold cross-validation repeated 10 times to reduce underfitting or overfitting and used the grid search method to optimize the model hyperparameters. The main metrics used to evaluate classification performance were accuracy and the area under the curve (AUC).

## Results

### Demographic data

A total of 157 patients with BD (55.4% male participants; mean age: 31.16 ± 14.25 years) and 544 patients with MDD (33.8% male participants; mean age: 21.15 ± 13.07 years) were included in the discovery cohort. A total of 119 patients with BD (51.26% men; mean age: 31.91 ± 11.68 years) and 95 patients with MDD (45.26% men; mean age: 37.63 ± 13.41 years) were included in the validation cohort. This study also included 273 HCs (34.1% men; mean age: 37.92 ± 9.22 years). All the participants received treatment, and the differences in medications (including antidepressants, antipsychotics, and mood stabilizers) between the BD and MDD groups were significant (Table 1). Detailed information on the medication use of the participants is provided in the Supplementary Table S1.

**Table 1:** Medication information of BD and MDD groups.

**Medications**		**BD (*n* = 157)**	**MDD (*n* = 544)**	***X* ^2^**	***P***
Antidepressants	Prescribed	49	508	288.530	<0.001
	Non-prescribed	108	36		
Antipsychotics	Prescribed	151	461	14.375	<0.001
	Non-prescribed	6	83		
Mood stabilizers	Prescribed	151	319	77.708	<0.001
	Non-prescribed	6	225		

BD, bipolar disorders; MDD, major depressive disorder.

### Peripheral non-enzymatic antioxidants at baseline in the BD, MDD, and HC groups

**Figure 1:**
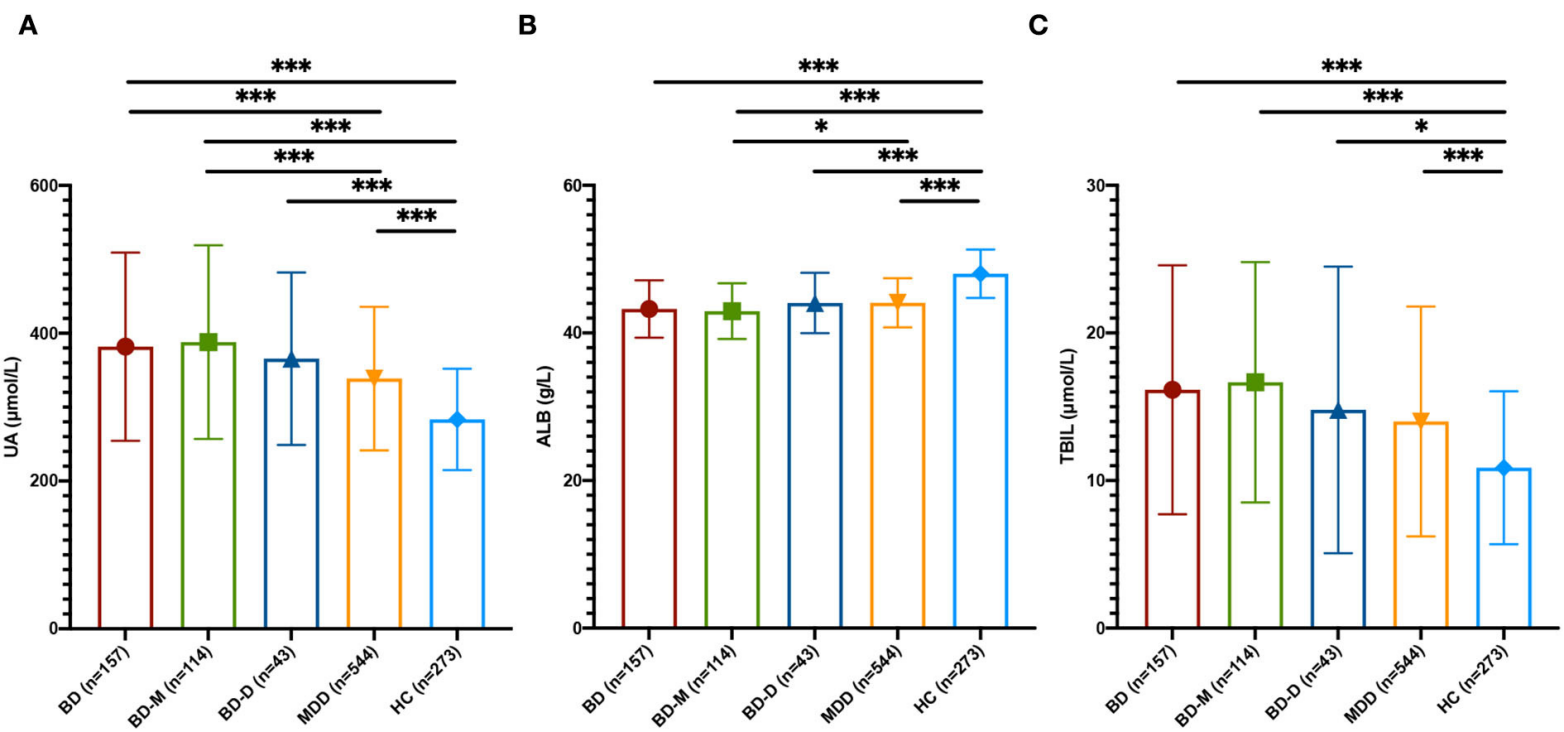
Peripheral non-enzymatic antioxidants at baseline among BD, MDD, and HC. **(A)** UA levels among BD, MDD, and HC groups. **(B)** ALB levels among BD, MDD, and HC groups. **(C)** TBIL levels among BD, MDD, and HC groups. UA, uric acid; ALB, albumin; TBIL, total bilirubin; BD, bipolar disorder; BD-M, bipolar disorder with mania/hypomania/mixed episode; BD-D, bipolar disorder with depression episode; MDD, major depressive disorder; HC, healthy control; *p < 0.05; ***p < 0.001.

At baseline, the differences in three non-enzymatic antioxidants among the BD, MDD, and HC groups were significant (UA, *F* = 23.512, *p* < 0.001; ALB, *F* = 131.385, *p* < 0.001; TBIL, *F* = 31.343, *p* < 0.001). The UA levels in the BD group were higher than those in the MDD group and HC group (*p* < 0.001), and the UA levels in the MDD group were also higher than those in the HC group (*p* < 0.001). The ALB levels of the BD group and MDD group were lower than those of the HC group (*p* < 0.001), while there were no significant differences in ALB levels between the BD group and MDD group (*p* = 0.179). The TBIL levels of the BD group and MDD group were higher than those of the HC group (*p* < 0.001), while the differences in TBIL levels between the BD group and MDD group were not significant (*p* = 0.565; Figure 1).

Then, we divided the BD group into the bipolar disorder with mania/hypomania/mixed episode (BD-M) subgroup and the bipolar disorder with depression episode (BD-D) subgroup. The results showed that UA levels in the BD-M subgroup were higher than those in the BD-D subgroup and MDD group (*p* < 0.001), but the differences in UA levels between the BD-D subgroup and the MDD group were not significant. ALB levels of the BD-M subgroup were higher than the MDD group (*P* = 0.043), while the differences between the BD-D subgroup and the MDD group were not significant. There were no significant differences in TBIL among the BD-M subgroup, BD-D subgroup, and MDD group (Figure 1).

In the validation cohort, similar results were found, while the UA levels of the BD-D subgroup were higher than those of the MDD group in the validation cohort. The detailed statistical results are shown in Supplemetary Tables S2–S4.

### Changes in three non-enzymatic antioxidants after treatment

**Figure 2:**
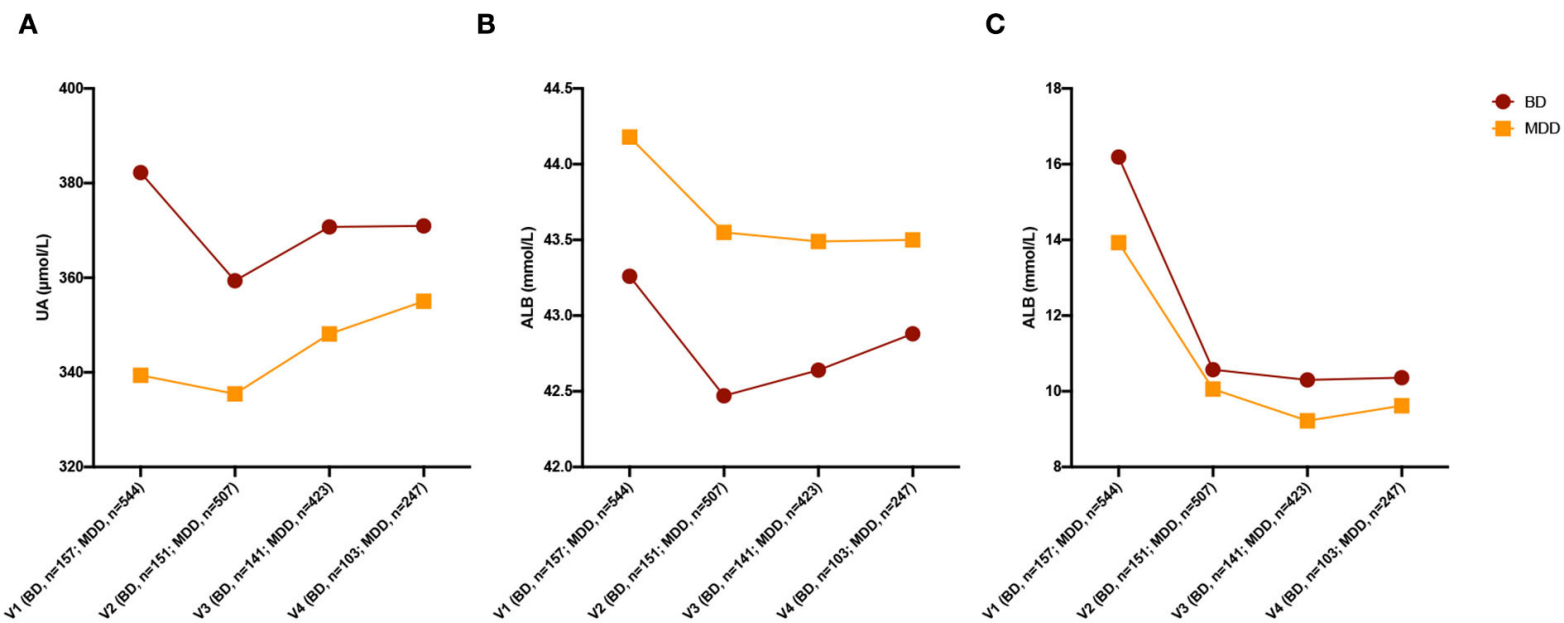
Changing of three non-enzymatic antioxidants after treatment. **(A)** Changing of UA levels after treatment. **(B)** Changing of ALB levels after treatment. **(C)** Changing of TBIL levels after treatment. UA, uric acid; ALB, albumin; TBIL, total bilirubin; BD, bipolar disorder; MDD, major depressive disorder; HC, healthy control.

The UA levels in the BD group decreased after treatment, while the UA levels in the MDD group increased after treatment. The changing trend of UA was significant (MDD and V1 as a reference, *Wald Chi-Square*_*diagnosis*_ = 4.244, *p* = 0.039, $WaldChi-Square_{diagnosis^{*}time}$ = 5.714, *p* = 0.017; age, sex, number of antidepressants, antipsychotics, and mood stabilizers were set as covariates). The ALB levels in the MDD group decreased after treatment, while there was no significant change in ALB levels in the BD group after treatment. The TBIL levels of both groups decreased after treatment. The changing trends of ALB and TBIL levels between the MDD group and the BD group were not significant (age, sex, number of antidepressants, antipsychotics, and mood stabilizers were set as covariates, Figure 2). The detailed statistical results are shown in Supplementary Tables S5–S7.

### Effectiveness of non-enzymatic antioxidants and clinical data in distinguishing between BD and MDD

**Figure 3:**
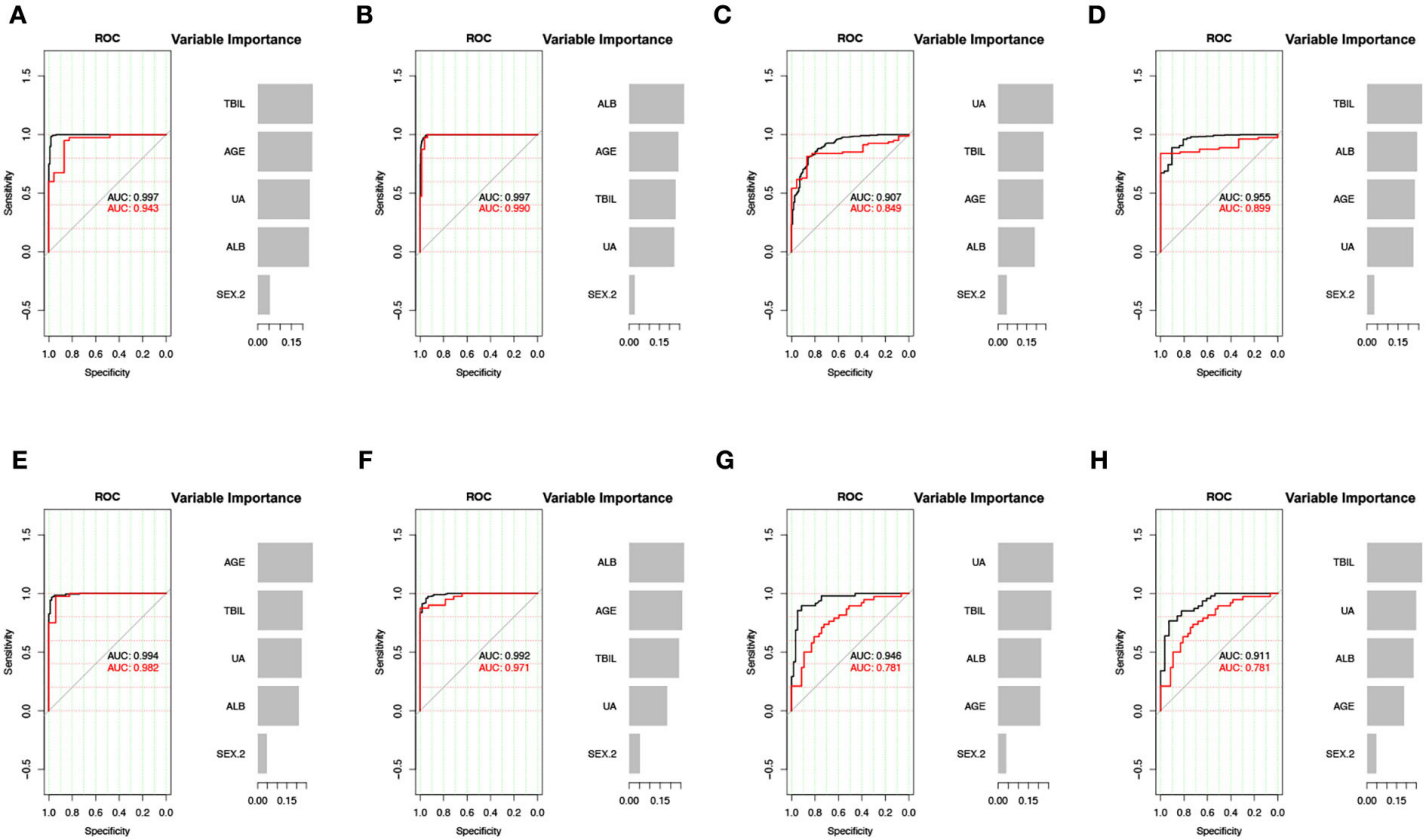
XGBoost model for the predictive effect of non-enzymatic antioxidants. **(A–D)** The result of discovery data. **(E–H)** The result of validation data. **(A,E)** BD vs. HC. **(B,F)** MDD vs. HC. **(C,G)** BD vs. MDD. **(D,H)** BD-D vs. MDD. UA, uric acid; ALB, albumin; TBIL, total bilirubin; BD, bipolar disorder; MDD, major depressive disorder; BD-D, bipolar disorder with depression episode; HC, healthy control; ROC, receiver operating characteristic curve; AUC, area under the curve.

First, we used age, sex, and three non-enzymatic antioxidants to distinguish between the mood disorders and HC groups. The results showed that the AUC of the XGBoost model for distinguishing between BD and HC groups was 0.943 (accuracy = 0.905, 95% CI = 0.804–0.964), and the AUC for distinguishing between MDD and HC groups was 0.990 (accuracy = 0.909, 95% CI = 0.843–0.954). Then, we used these data to distinguish between BD and MDD, as well as between BD-D and MDD. The AUC of the XGBoost model for distinguishing between BD and MDD groups was 0.849 (accuracy = 0.808, 95% CI = 0.719–0.878), and the AUC for distinguishing between BD-D subgroup and MDD group was 0.899 (accuracy = 0.891, 95% CI = 0.856–0.919). Finally, we used an independent cohort, including 119 subjects with BD, and 95 subjects with MDD, as a validation cohort to verify our results. Detailed information on the validation cohort has been published in our previous study (15). The above results were confirmed. The AUC of the XGBoost model in validation cohort for distinguishing between BD and HC groups was 0.982 (accuracy = 0.947, 95% CI = 0.854–0.989), the AUC for distinguishing between MDD and HC was groups 0.971 (accuracy = 0.889, 95% CI = 0.774–0.958), the AUC for distinguishing between BD and MDD groups was 0.781 (accuracy = 0.706, 95% CI = 0.597–0.800), the AUC for distinguishing between BD-D and MDD groups was 0.781 (accuracy = 0.633, 95% CI = 0.499–0.754). The most important feature for distinguishing between BD and MDD was UA in both the discovery cohort and validation cohort (Figure 3).

The XGBoost model showed better classifying ability than the logistic regression model (Table 2).

**Table 2:** AUC of two prediction models.

	**BD vs. HC** **(*n* = 157 vs. *n* = 273)**	**MDD vs. HC** **(n = 544 vs. *n* = 273)**	**BD vs. MDD (*n* = 157 vs. *n* = 544)**	**BD-D vs. MDD** **(*n* = 43 vs. *n* = 544)**
**Discovery cohort**				
Logistic regression	0.971	0.960	0.729	0.710
XGBoost	0.943	0.990	0.849	0.899
	**BD vs. HC** **(*****n*** = **119 vs**. ***n*** = **273)**	**MDD vs. HC** **(*****n*** = **95 vs**. ***n*** = **273)**	**BD vs. MDD** **(*****n*** = **119 vs**. ***n*** = **95)**	**BD-D vs. MDD** **(*****n*** = **55 vs**. ***n*** = **95)**
**Validation cohort**				
Logistic regression	0.966	0.963	0.759	0.698
XGBoost	0.982	0.971	0.781	0.781

BD, bipolar disorder; MDD, major depressive disorder; BD-D, bipolar disorder with depression episode; HC, healthy control; AUC, area under the curve.

## Discussion

In this study, we compared three peripheral non-enzymatic antioxidants among patients with BD, MDD, and HCs. The result showed that the differences in all 3 indices of interest among 3 groups were significant; the UA and TBIL levels of the BD and MDD groups were higher than those of the HC group, while their ALB levels were lower than those of the HC group. These results indicated that mood disorders were associated with dysfunction of the peripheral antioxidation system.

Mitochondria are able to reduce deleterious oxidation, but this ability is not sufficient to neutralize all oxidative stress. Therefore, antioxidants are necessary to prevent excessive oxidative damage. Enzymatic antioxidants can inhibit the genesis of peroxide and remove excessive reactive oxygen species (16). Non-enzymatic antioxidants also play a key role in the antioxidant system and can chelate transition metals and interact with reactive oxygen species by breaking free radical chain reactions (17). There are many non-enzymatic antioxidants in plasma, such as UA, ALB, TBIL, zinc, tocopherol, ascorbate, and retinol. In addition to the three non-enzymatic antioxidants, other compounds also have important biological functions. For instance, retinol is essential for embryonic development, especially for the development of the brain, meanwhile, the ascorbate, as a neuroprotective compound, is highly concentrated in the brain and regulates the function of neurons and synapses (12, 18, 19). Abnormal levels of these antioxidants in mood disorders were widely discussed in previous studies, and some meta-analyses were conducted, which showed that their results were consistent with our findings (10, 11). However, many non-enzymatic antioxidants are trace elements and are not routinely measured in the clinic. In contrast, the three indices detected in this study, which accounted for approximately 85% of the antioxidant capacity of plasma, could represent the level of peripheral antioxidants. In addition, they are routinely examined during hospitalization and are easy to obtain (14).

Uric acid, as a selective antioxidant, can scavenge reactive oxygen or nitrogen and prevent the erythrocyte membrane from lipid peroxidation by reacting with peroxides (20). Moreover, it is the end-product of the purinergic system, which is involved in the pathophysiology of mental disorders *via* influencing cell proliferation, neuronal differentiation, and neuroglial cell inflammation (21, 22). UA is also associated with sleep, cognition, appetite, social interaction, etc. (23, 24). In this study, UA levels in the BD group were found to be higher than those in the MDD group, which suggested that BD might have a more severe imbalance of redox homeostasis and that UA might be a potential biomarker to distinguish between BD and MDD. Then, we divided the BD group into BD-M and BD-D subgroups. The UA levels of BD-M were markedly higher than those of MDD, while the differences in UA between BD-D and MDD were not significant, implying that UA might be a status indicator for BD. Therefore, we checked the changing trend of UA, which decreased after treatment in the BD group, in contrast, UA increased after treatment in the MDD group, which supported UA as a potential biomarker to distinguish BD from MDD. Previous studies also showed that the UA of the BD group was higher than MDD and HC groups (25–27), and the UA levels of BD-M were higher than those of BD-D and euthymic stage (28–30), while the results regarding UA in MDD were inconsistent. Several studies reported that MDD was associated with lower UA levels than HC and BD (31), while there was a study that showed no significant differences in UA between MDD and HC groups (32). This study showed that the UA levels of MDD were higher than those of HC, which might result from the heterogeneity of the participants. This study aimed to explore peripheral antioxidants of mood disorders in a real-world setting, and we set sex, age, and medication use as covariates to control for confounding factors. Although the diet might significantly affect the level of the UA, all the participants were hospitalized and their diets were essentially the same. In our previous study, age was negatively associated with UA levels, and the age of the MDD group was lower than that of HC, which might be a reason for higher levels of UA in MDD than in HC (15). The consistent results of UA in BD indicated that UA was a reliable biomarker for BD.

Albumin is an endogenous antioxidant with a radical scavenging function, binding metal ions, and responsible for reactive oxygenated radical species. Furthermore, ALB, which plays a role in the inflammation and the immune system, has also been demonstrated to be involved in the pathogenesis of mental disorders. Bilirubin plays a role in antioxidative mechanisms by efficiently scavenging peroxyl radicals and acting as a chain-breaking antioxidant. Besides the antioxidant ability of TBIL, it has toxic effects on the brain, and a great deal of evidence indicates that it is related to cognitive function. Abnormal levels of TBIL were also found in mental disorders (33). Differences in ALB and TBIL between BD and MDD groups were neither significant nor were the changing trends of these two indices after treatment. However, the ALB in the BD-M subgroup was lower than those in the MDD group, implying that patients with mania episodes might have more severe dysfunction of the antioxidation system than patients with MDD.

Previous studies indicated that the antidepressants (such as escitalopram), mood stabilizers (such as lithium and valproate), and antipsychotics (such as olanzapine and clozapine) may have neuroprotective effects against oxidative stress (34–40), while antioxidative effects differed among different types of medications, and the conclusion was not consistent (41, 42). In this study, we set the numbers of antidepressants, mood stabilizers, and antipsychotics as covariates when detecting the changing trend after treatment to avoid confounding factors, and the results were still significant.

Misdiagnosis is common in BD since it often starts with a depression episode (2). It leads to inappropriate treatment, switching to mania/hypomania, and repeated attacks (43). Based on the above findings, non-enzymatic antioxidants might be potential biomarkers. We applied age, sex, and three non-enzymatic antioxidants to distinguish between BD and MDD. The AUC was 0.849, and UA was the most important feature for distinguishing between BD and MDD, which was used to confirm the results.

Some researchers believe that both MDD and BD belong to the same mood disorder spectrum; they used novel terms such as bipolar spectrum disorders to describe these conditions (44). Some researchers hold different views, and they attempted to distinguish between patients with BD and MDD by their personal characteristics using a “softer bipolar spectrum,” including the onset age, temperament, and response to antidepressants, and their concepts were validated by several studies (45–48). In addition, in contrast to the fourth version of the Diagnostic and Statistical Manual of Mental Disorders (DSM-IV), DSM-5 divides mood disorders into two chapters (bipolar disorders and depressive disorder), which confirms that BD is distinct from MDD.

There are some strengths and limitations to our study. The first advantage was the real-world measures used in the study that increased the clinical transferability of our results. Furthermore, the study also analyzed the changes in peripheral non-enzymatic antioxidants after treatment. The third advantage is that we applied a machine learning model, namely, the XGBoost model, to explore the predictive effect of non-enzymatic antioxidants, and the results showed that machine learning could improve classification ability. Nevertheless, some limitations should be discussed. First of all, the retrospective design of the study did not allow us to assess the severity of symptoms; in addition, the diagnosis could not be confirmed because of the short-observation period. The second limitation was, although all the participants were inpatients who received uniform diets provided by the hospital, we did not take into account certain confounding factors that may influence the levels of peripheral antioxidants, such as smoking and body mass index into account. Finally, we explored the change after treatment. Although we set the numbers of medications as covariates to prevent them from acting as confounders, the types of medications were too complex, so the detailed types of medications were not taken into account. In the future, a rigorously designed head-to-head randomized clinical trial should be conducted to explore the antioxidative effects of different medications.

In conclusion, the dysfunction of the peripheral non-enzymatic antioxidant system might be involved in the pathogenesis of mood disorders, and UA might be used as a potential biomarker to distinguish between BD and MDD.

## Data availability statement

The raw data supporting the conclusions of this article will be made available by the authors, without undue reservation.

## Ethics statement

The studies involving human participants were reviewed and approved by the Clinical Research Ethics Committee of Shandong Mental Health Center. The patients/participants provided their written informed consent to participate in this study.

## Author contributions

YG, ZL, GX, and WY designed the research. ZL, ZK, XF, and WC extracted and cleaned the data. ZL, YZ, and YS performed the statistical analysis. ZL wrote the original draft. YG, GX, and WY reviewed and edited the final manuscript. All authors contributed to and have approved the final manuscript.

## Funding

This study was supported by the National Natural Science Foundation of China (81825009); National Key R&D Program of China (2021YFF1201103); Academy of Medical Sciences Research Unit (2019-I2M-5-006); Chinese Institute for Brain Research at Beijing (2020-NKX-XM-12); PKUHSC-KCL Joint Medical Research (BMU2020KCL001) (WY). This study was also supported by Shandong Province Key R&D Program (Science and Technology Demonstration Project) Project (2021SFGC0504) (YG). This study was also supported by the Innovation Fund for Outstanding Doctoral Students of Peking University Health Science Center (ZL).

## Conflict of interest

The authors declare that the research was conducted in the absence of any commercial or financial relationships that could be construed as a potential conflict of interest.

## Publisher's note

All claims expressed in this article are solely those of the authors and do not necessarily represent those of their affiliated organizations, or those of the publisher, the editors and the reviewers. Any product that may be evaluated in this article, or claim that may be made by its manufacturer, is not guaranteed or endorsed by the publisher.
