# Supplementary material for: Peripheral non-enzymatic antioxidants as biomarkers for mood disorders: Evidence from a machine learning prediction model
Source: Front Psychiatry. 2022 Nov 7;13:1019618. doi: 10.3389/fpsyt.2022.1019618 (PMC9676245; doi:10.3389/fpsyt.2022.1019618)
Supplement: Supplementary file 1 [file Data_Sheet_1.docx]

**Supplementary Materials**

**Supplementary Methods**

**Subjects and participants in validation cohort**

The study protocol was approved by the Clinical Research Ethics Committee of Shandong Mental Health Center and is compliant with the Code of Ethics of the World Medical Association (Declaration of Helsinki). Informed written consent was obtained from all participants or their legal guardians after a complete and extensive description.

The study was conducted at Shandong Mental Health Center, from May 2018 to May 2019, inpatients and outpatients aged from 18-60 years with the Diagnostic and Statistical Manual of Mental Disorders, fifth edition (DSM-5) diagnosis of BD or UD were recruited. Furthermore, healthy individuals with no family history of psychiatric disorders were enrolled in the study as the control group.

Inclusion criteria for patients: 1) meet the bipolar disorder or major depressive disorder criteria based on DSM-5; 2) age 18-60 years, Han Chinese; 3) understand research content and provide written informed consent.

Inclusion criteria for healthy controls (HC): 1) without any mental disorders and family history of mental disorders; 2) age 18-60 years, Han Chinese; 3) HAMD-17＜7, YMRS＜6; 4) understand research content and provide written informed consent.

The exclusion criteria for all participants were as follows: 1) Combined with organic brain diseases or brain trauma; 2) Hypertension, diabetes, gout or liver, kidney, biliary, and other physical diseases or abnormal renal and liver function; 3) Combined with other mental disorders; 4) Positive in urine pregnancy test or lactating females; 5) Modified electroconvulsive therapy treatment within 4 weeks, or long-acting antipsychotics treatment within 6 months; 6) Taking antioxidants or neurotrophic drugs within 12 weeks before and during enrollment.

All participants received an interview by a psychiatric postgraduate (Zhe Lu), the diagnosis was confirmed by at least two experienced psychiatrists based on DSM-5.

**Supplementary Tables**

**Table S1. Medication information**

| Medications | BD (n=157) | MDD (n=544) |
| --- | --- | --- |
| Antidepressants |  |  |
| Non-prescribed | 108 | 36 |
| One type | 39 | 328 |
| Two types | 9 | 169 |
| Three types | 1 | 11 |
| Escitalopram | 29 | 359 |
| Citalopram | 1 | 9 |
| Sertraline | 5 | 45 |
| Fluoxetine | 1 | 18 |
| Fluvoxamine | 2 | 18 |
| Paroxetine | 5 | 21 |
| Duloxetine | 3 | 40 |
| Venlafaxine | NA | 55 |
| Trazodone | 1 | 19 |
| Agomeladine | 1 | 28 |
| Bupropion | 8 | 13 |
| Valteoxetine |  | 2 |
| Mirtazapine | 3 | 70 |
| Mianserin | 1 | 15 |
| Antipsychotics |  |  |
| Non-prescribed | 6 | 83 |
| One type | 96 | 371 |
| Two types | 49 | 83 |
| Three types | 6 | 17 |
| Olanzapine | 67 | 154 |
| Quetiapine | 73 | 152 |
| Aripiprazole | 12 | 121 |
| Risperidone | 15 | 12 |
| Ziprasidone | 11 | 27 |
| Amisupiride | 11 | 33 |
| Sulpiride | NA | 14 |
| Clozapine | 1 | 1 |
| Perphenazine | 6 | 5 |
| Paliperidone | 4 | 28 |
| Lurasidone | 10 | NA |
| Haloperidol | 9 | 7 |
| Perospirone | NA | 1 |
| Mood stabilizers |  |  |
| Non-prescribed | 6 | 225 |
| One type | 60 | 254 |
| Two types | 81 | 61 |
| Three types | 10 | 4 |
| Lithium carbonate | 98 | 59 |
| Valproate | 94 | 178 |
| Carbamazepine | 1 | 1 |
| Oxcarbazepine | 22 | 38 |
| Lamotrigine | 37 | 111 |

BD, bipolar disorders; MDD, major depressive disorder.

**Table S2. Peripheral non-enzymatic antioxidants at baseline among BD, MDD and HC.**

|  | BD (n=157) |  | MDD (n=544) |  | HC (n=273) |  | *F* | *P* |
| --- | --- | --- | --- | --- | --- | --- | --- | --- |
|  | Mean | SD | Mean | SD | Mean | SD |  |  |
| UA | 381.80 | 127.30 | 338.42 | 97.10 | 283.41 | 68.61 | 34.550 | <0.001 |
| *Post-hoc* test (*Bonforroni* test): BD>MDD>HC | | | | | | | | |
| ALB | 43.25 | 3.88 | 44.09 | 3.32 | 48.02 | 3.28 | 193.83 | <0.001 |
| *Post-hoc* test (*Bonforroni* test): BD, MDD<HC | | | | | | | | |
| TBIL | 16.15 | 8.43 | 13.99 | 7.79 | 10.87 | 5.18 | 31.343 | <0.001 |
| *Post-hoc* test (*Bonforroni* test): BD, MDD>HC | | | | | | | | |

Covariates: age, sex.

UA, uric acid; ALB, albumin; TBIL, bilirubin; BD, bipolar disorder; MDD, major depressive disorder; HC, healthy control.

**Table S3. Peripheral non-enzymatic antioxidants at baseline among BD-M, BD-D, MDD and HC.**

|  | BD-M (n=114) |  | BD-D (n=43) |  | MDD  (n=544) |  | HC  (n=273) |  | *F* | *P* |
| --- | --- | --- | --- | --- | --- | --- | --- | --- | --- | --- |
|  | Mean | SD | Mean | SD | Mean | SD | Mean | SD |  |  |
| UA | 387.98 | 131.1 | 365.54 | 116.61 | 338.42 | 97.10 | 283.41 | 68.61 | 23.512 | <0.001 |
| *Post-hoc* test (*Bonforroni* test): BD-M > BD-D, MDD > HC | | | | | | | | | | |
| ALB | 42.95 | 3.77 | 44.06 | 4.1 | 44.09 | 3.32 | 48.02 | 3.28 | 131.385 | <0.001 |
| *Post-hoc* test (*Bonforroni* test): BD-M < MDD < HC | | | | | | | | | | |
| TBIL | 16.66 | 8.14 | 14.79 | 9.712 | 13.99 | 7.79 | 10.87 | 5.18 | 31.343 | <0.001 |
| *Post-hoc* test (*Bonforroni* test): BD-M, BD-D, MDD > HC | | | | | | | | | | |

Covariates: age, sex.

UA, uric acid; ALB, albumin; TBIL, total bilirubin; BD-M, bipolar disorder with mania/hypomania/mixed episode; BD-D, bipolar disorder with depression episode; MDD, major depressive disorder; HC, healthy control.

**Table S4. Peripheral non-enzymatic antioxidants among BD-M, BD-D, MDD and HC in validation cohort.**

|  | BD  （n=119） | BD-M（n=64） | BD-D（n=55） | MDD  （n=95） | HC  (n=273) | F | P |
| --- | --- | --- | --- | --- | --- | --- | --- |
| UA | 354.02±88.75 | 367.84±92.92 | 337.93±81.54 | 282.13±77.98 | 283.41±68.61 | 31.529 | <0.001 |
| *Post-hoc* test (*Bonforroni* test): BD>MDD,HC; BD-M>BD-D>MDD,HC (*F_sub*=26.799, *P_sub*<0.001) | | | | | | | |
| ALB | 43.10±3.59 | 43.03±3.54 | 43.18±3.67 | 42.78±4.01 | 48.02±3.28 | 152.402 | <0.001 |
| *Post-hoc* test (*Bonforroni* test): BD,MDD<HC; BD-M,BD-D,MDD<HC (*F_sub*=101.413, *P_sub*<0.001) | | | | | | | |
| TBIL | 17.14±6.84 | 17.46±7.25 | 16.77±6.38 | 17.62±6.88 | 10.87±5.18 | 70.198 | <0.001 |
| *Post-hoc* test (*Bonforroni* test): BD,MDD>HC; BD-M,BD-D,MDD>HC (*F_sub*=47.086, *P_sub*<0.001) | | | | | | | |

Covariates: age, sex.

UA, uric acid; ALB, albumin; TBIL, total bilirubin; BD-M, bipolar disorder with mania/hypomania/mixed episode; BD-D, bipolar disorder with depression episode; MDD, major depressive disorder; HC, healthy control.

**Table S5. Changing of UA levels after treatment.**

|  | Diagnosis | Mean | SDE | 95%CI | |
| --- | --- | --- | --- | --- | --- |
| V4 | BD | 370.97 | 8.91 | 353.50 | 388.44 |
|  | MDD | 355.05 | 5.66 | 343.96 | 366.13 |
| V3 | BD | 370.75 | 8.60 | 353.90 | 387.60 |
|  | MDD | 348.12 | 4.90 | 338.51 | 357.73 |
| V2 | BD | 359.36 | 8.12 | 343.44 | 375.27 |
|  | MDD | 335.46 | 4.52 | 326.60 | 344.31 |
| V1 | BD | 382.25 | 4.52 | 361.46 | 403.04 |
|  | MDD | 339.39 | 4.54 | 330.49 | 348.29 |

UA, uric acid; BD, bipolar disorder; MDD, major depressive disorder.

**Table S6. Changing of ALB levels after treatment.**

|  | Diagnosis | Mean | SDE | 95%CI | |
| --- | --- | --- | --- | --- | --- |
| V4 | BD | 42.88 | 0.35 | 42.20 | 43.56 |
|  | MDD | 43.50 | 0.20 | 43.12 | 43.89 |
| V3 | BD | 42.64 | 0.33 | 41.99 | 43.29 |
|  | MDD | 43.49 | 0.15 | 43.20 | 43.78 |
| V2 | BD | 42.47 | 0.30 | 41.88 | 43.06 |
|  | MDD | 43.55 | 0.14 | 43.26 | 43.83 |
| V1 | BD | 43.26 | 0.33 | 43.63 | 43.83 |
|  | MDD | 44.18 | 0.16 | 43.87 | 44.49 |

ALB, albumin; BD, bipolar disorder; MDD, major depressive disorder.

**Table S7. Changing of TBIL levels after treatment.**

|  | Diagnosis | Mean | SDE | 95%CI | |
| --- | --- | --- | --- | --- | --- |
| V4 | BD | 10.36 | 0.43 | 9.53 | 11.2 |
|  | MDD | 9.62 | 0.35 | 8.93 | 10.31 |
| V3 | BD | 10.30 | 0.38 | 9.55 | 11.04 |
|  | MDD | 9.22 | 0.25 | 8.74 | 9.71 |
| V2 | BD | 10.57 | 0.46 | 9.67 | 11.48 |
|  | MDD | 10.06 | 0.29 | 9.49 | 10.62 |
| V1 | BD | 16.19 | 0.68 | 14.86 | 17.52 |
|  | MDD | 13.93 | 0.37 | 13.20 | 14.65 |

TBIL, bilirubin; BD, bipolar disorder; MDD, major depressive disorder.
